# Mediating role of coping strategies in the relationship between death anxiety and insomnia among patients with non-communicable diseases: a gender perspective

**DOI:** 10.1038/s41598-025-99348-3

**Published:** 2025-05-02

**Authors:** Arslan Khalid, Abdul Sattar Ghaffari, Khizra Iqbal, Yonggang Su

**Affiliations:** 1 School of Foreign Languages and Literature, Shandong University, Jinan, 250100 Shandong China; 2 Biostatistician, Department of ORIC, Shifa Tameer-e-Millat University, Islamabad, Pakistan; 3 Department of Psychiatry and Behavioral Sciences, Nishtar Medical University and Hospital, Multan, Punjab Pakistan

**Keywords:** Non-communicable diseases, Coping strategies, Death anxiety, Insomnia, Gender, Psychology, Health care

## Abstract

Non-communicable diseases (NCDs) such as cancer, diabetes, and cardiovascular disorders pose significant challenges to global health, often leading to chronic conditions that can profoundly impact patients’ mental and emotional well-being. This study aims to explore the mediating role of coping strategies in the relationship between death anxiety and Insomnia among patients with NCDs, with a particular focus on gender differences. By examining these dynamics, the research seeks to contribute to more personalized and effective psychological support for patients facing the dual burden of chronic illness and mental health challenges. Cross-sectional research design by using survey method. Death Anxiety Scale, Athens Insomnia Scale, and Brief Coping Inventory were used to gauge the respective variables. A sample of (*n* = 264) diagnosed patients with non-communicable diseases (diabetes, hypertension, cardiovascular diseases, and cancer) was selected through purposive sampling. Findings revealed a positive correlation between death anxiety and Insomnia and a negative association with coping strategies in patients with non-communicable diseases. The use of coping strategies as a mediator showed the indirect pathway, which fully mediates the relationship between death anxiety and Insomnia in male (0.57, *p* < .05) and female (0.663, *p* < .05) respondents. Furthermore, the prevalence of death anxiety and Insomnia was higher in females as compared to males. The use of problem-focused coping strategies was higher in males whereas emotion-focused coping was more in females. It is concluded that the use of coping strategies mediates a significant relationship between death anxiety and Insomnia. Patients with non-communicable diseases experience intrusive thoughts of death anxiety in Insomnia, and coping techniques contribute to a more balanced and resilient approach to handling the difficulty associated with death anxiety and Insomnia. This study will be helpful for the respective specialists to manage the variability of symptoms and maintain the complaints their patients come up with during their treatment.

Non-communicable diseases (NCDs) pose a significant global health burden, accounting for 71% of all deaths worldwide. Diseases NCDs such as cardiovascular diseases, cancer, chronic respiratory diseases, and diabetes are leading causes of morbidity and mortality worldwide^1^. Non-communicable diseases are defined as diseases that are caused by unhealthy behaviors. Worldwide, these diseases are the leading cause of death, particularly in low and middle-income countries. In Pakistan, as statistically mentioned, approximately 50% of Pakistan’s population suffers from one or more of these non-communicable diseases, such as diabetes, hypertension, cardiac disease, and cancer^2^. Patients with NCDs often experience significant psychological distress, including death anxiety and Insomnia, which can adversely impact their quality of life^3^.

According to Lazarus and Folkman’s Transactional Model of the coping process how individuals appraise and respond to psychological stressors that come from the external environment, the adaptive and maladaptive coping mechanism leads to an impact on psychological and physiological outcomes such as insomnia. Coping strategies are individuals’ cognitive and behavioral efforts to manage stress and mitigate the impact of adverse events^4^. Effective coping strategies can alleviate the psychological distress associated with death anxiety and reduce Insomnia symptoms^5^. Coping strategies that target the underlying anxiety can have a positive impact on sleep patterns and play a significant role in the management of patient concerns. Coping is the continuous effort of a given individual to change cognition and behavior to cope with demands considered stressful and beyond the reach of personal resources. Previous evidence suggested that coping styles play a critical mediating role in psychological stress. Individual coping styles can affect both the properties and strength of response to stress and anxiety^6^.

Patients with NCDs often face increased mortality concerns, which can lead to death anxiety. Death is defined as a biological, existential, personal, and socio-cultural phenomenon of which only humans among all beings are aware. Death anxiety, defined as the fear of death or dying, is particularly prevalent among individuals with life-threatening conditions^2^. Death anxiety may contribute to Insomnia, as worrying about mortality can make it difficult to sleep. Insomnia is one of the most prevalent sleep disorders^7^, which can be characterized as difficulty initiating or maintaining sleep, resulting in fatigue and affecting routine life functioning. American Psychiatric Association defines Insomnia as a prominent complaint of dissatisfaction with sleep quality or quantity associated with difficulty in initiating and maintaining sleep along with the earlier awakening with the inability to return to sleep. High prevalence of Insomnia in a variety of medical and psychiatric conditions. In addition, there is a significant relationship between death anxiety and a lot of subjective states, including Insomnia.

Insomnia, characterized by difficulty falling asleep, staying asleep, or waking up too early, is a prevalent sleep disorder among individuals with chronic illnesses^8,9^. It can lead to fatigue, impaired cognitive function, and reduced quality of life^10,11^. Furthermore, Insomnia has been linked to an increased risk of mortality in patients with NCDs^12^.

Moreover, gender differences in coping strategies, death anxiety, and Insomnia have been reported, suggesting the need for a gender-specific approach to understanding and addressing these issues^13,14^. However, few studies have examined the mediating role of coping strategies in the relationship between death anxiety and Insomnia among patients with NCDs from a gender perspective. Gender differences in coping strategies and their effectiveness have been well-documented in the literature. Males and females often employ different coping mechanisms, which can influence the psychological outcomes associated with chronic illness^15^. Women are more likely to use emotion-focused coping strategies, such as seeking social support, while men are more inclined to use problem-focused coping strategies^16^. These gender-specific coping patterns may modulate the impact of death anxiety on Insomnia, necessitating a gender perspective in this research^17,18^.

Non-communicable diseases are observed to be more prevalent nowadays, especially in Pakistani culture, than it was before. Though increased attention has been given to the comorbidity of non-communicable diseases and psychiatric disorders, very little research has been done on it, especially from a gender perspective. Present research holds significant value by examining the mediating role of coping strategies in the relationship between death anxiety and Insomnia offering a gender-based analysis. The findings will provide empirical evidence for contribution to the field of psychological research by assessing whether coping strategies mitigate the negative effects of death anxiety on sleep disturbances differently in males and females. Additionally, it will offer valuable implications for mental health professionals, enabling them to design gender-sensitive interventions that address death anxiety and improve coping mechanisms to reduce Insomnia symptoms among patients with non-communicable diseases.

We hypothesized that death anxiety would be positively associated with Insomnia and negatively associated with the use of coping strategies. Death anxiety will have a direct effect on Insomnia mediated by using coping strategies in males and females. Death anxiety and Insomnia symptoms will be more prevalent in females as compared to males. The use of coping strategies will be more effective in male patients as compared to females. Death anxiety, Insomnia, and use of coping strategies will differ in terms of demographic variables (residential area, age group).

## Methods

### Participants and procedure

A cross-sectional survey was conducted from March to July 2024 in Pujab Province, Pakistan (population: 120 million). Participants were *n* = 264 (142 males and 122 females, age range 18–60 years) selected through purposive sampling from Nishtar Hospital and Multan Civil Hospital, as these hospitals are hubs for all medical and psychiatric patients. Based on inclusion and exclusion criteria, diagnosis of non-communicable diseases (diabetes, hypertension, cardiovascular diseases cancer, and others) or admission to the hospital for any of the following surgeries along with the presence of psychiatric symptoms of death, anxiety, or disorder make up the inclusion criteria for the study, whereas exclusion criteria include the presence of any other comorbid medical or psychiatric disorder or the symptoms of both death anxiety and Insomnia altogether. In addition, patients under 15 and those in very critical condition (on ventilators, etc.) were excluded. Lastly, addictive patients were not taken for the present study.

A booklet consisting of informed consent, a demographics sheet, and a questionnaire were given to the participants. After obtaining informed consent from the participants, they were debriefed about the study. It was ensured to the participants that their confidentiality and privacy would be maintained. Each participant completed the questionnaire within 20 min under the supervision of the researcher. A total of 300 participants, appropriate responses were obtained from 264 participants as 36 participants did not complete the questionnaires or voluntarily withdrew, yielding a response rate were 88.0%, including 53.8% male and 46.2% female.

### Measures

#### Death anxiety scale

Death anxiety was measured using the DAS, a 15-item true-false self-report scale aimed to determine the extent to which a person undergoes death anxiety. The responses can range from 0 to 15, with the higher the score, the more severe the death anxiety. It reported 0.83 test-retest reliability and 0.76 internal consistency for the respective scale^19^.

#### Athens insomnia scale (AIS)

The AIS is also a self-report instrument for quantifying Insomnia’s severity, a sleep disorder based on the ICD-10 criteria. But it also meets the criteria for Insomnia. The higher the score, the more severe the insomnia. The Cronbach’s value for internal consistency of the scale was 0.90, whereas significantly high the test-retest reliability was determined as 0.90 at a 1-week interval^20^.

#### Brief Cope

The Brief Cope comprises 28 items divided into 14 subscales, each representing a distinct coping strategy. These strategies include active coping, planning, positive reframing, acceptance, humour, religion, emotional support, instrumental support, self-distraction, denial, venting, substance use, behavioral disengagement, and self-blame. Participants rate each item on a 4-point Likert scale, ranging from 1 (“I haven’t been doing this at all”) to 4 (“I’ve been doing this a lot”). A high score indicates more use of coping strategies. The reliability of the scale is 0.90^21^.

### Data analysis

**Fig. 1:**
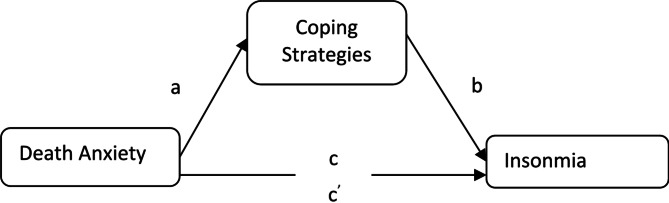
Based on Lazarus and Folkman’s Cognitive appraisal Theoretical model of the mediating role of coping strategies in the association between death anxiety and Insomnia. In this theoretical model (**a**) associations of death anxiety with coping strategies; (**b**) association between coping strategies with insomnia; (**c**) direct associations of death anxiety with insomnia; (**c’**) associations of death anxiety with Insomnia after adding coping strategies as a mediator.

For analyzing the data both descriptive and inferential statistical analysis Pearson correlation, mediation analysis, t-tests, and one-way ANOVA were conducted. In the mediation analysis, the value of *a* denotes the association of death anxiety and coping strategies, and path *b* denotes the association of coping strategies with Insomnia. Path *c* denotes the association between death anxiety and Insomnia (Fig. 1).

### Ethics

The study was approved by the Institutional Ethical Review Board of Nishtar Medical University, Multan Reference No: 3339, dated 02-02-2024.

*Theoretical framework*. The theoretical framework in this study is as follows:

## Results

Table 1, presents the frequencies and percentages of the demographic variables and main study variables.

**Table 1:** Descriptive statistics for study variables.

Variables	Category	Frequency (%)/Mean ± SD
Gender	Male	142 (53.8)
	Female	122 (46.2)
Age	Up to 25 years	86 (32.6)
	25–35 years	88 (33.3)
	35–45 years	60 (22.7)
	Above 45 years	30 (11.4)
Area	Urban	170 (64.4)
	Rural	94 (35.6)
Education	Illiterate	40 (15.2)
	Matric	40 (15.2)
	Intermediate	36 (13.6)
	Undergraduate	47 (17.8)
	Graduate	49 (18.6)
	Postgraduate	52 (19.7)
Non-communicable disease	Cardiovascular disease	110 (41.7)
	Diabetes	85 (32.2)
	Cancer	40 (15.2)
	Hypertension	20 (7.6)
	Others	9 (3.4)
Death anxiety	-	15.26 ± 3.19
Insomnia	-	12.10 ± 3.08
Coping strategies	-	70.29 ± 5.83

Table 2, indicates the correlations between death anxiety, Insomnia, and coping strategies. The findings reveal that death anxiety significantly and positively correlated with Insomnia (*r* = .648) whereas it significantly and negatively correlated with coping strategies (*r* = -.817). Similarly, Insomnia and coping strategies are significantly and negatively correlated with each other (*r* = -.814).

**Table 2:** Correlation between death anxiety, insomnia, and coping strategies.

Variables	M ± SD	1	2	3
Death anxiety	15.26 ± 3.19	1		
Insomnia	12.10 ± 3.08	0.648**	1	
Coping strategies	70.29 ± 5.83	− 0.817**	− 0.814**	1

**significant at the 0.01 level (2-tailed).

The results emphasize the importance of addressing emotional, cognitive, and behavioral aspects in mental health care to improve overall well-being and reduce the burden of related psychological issues like insomnia. A Study^22^ similarly revealed a significant positive association between death anxiety and Insomnia. Findings are also consistent with our study results as a negative association of death anxiety with coping strategies was found. Another study also found an inverse correlation between death anxiety and coping strategies^23^. The study conducted^24^ also aligns with our findings that patients suffering from cancer disease showed a connection between death anxiety and Insomnia. Another study showed similar findings that stress and anxiety are positively associated with sleep quality and a negative relationship was found with coping style as a positive way of thinking patterns improve sleep quality and reduce anxiety among individuals^25^.

**Table 3:** Regression analysis, using coping strategies as a mediator and insomnia as an outcome.

Predictors	Path coefficients				(a * b)/c (95% CI)	*R* ^2^
	a	b	c	c^′^		
Male						
Death anxiety	-1.454***	− 0.452***	0.571***	− 0.086	0.657 (0.537, 0.801)	0.655
Female						
Death anxiety	-1.500***	− 0.444***	0.663***	− 0.002	0.666 (0.535, 0.833)	0.657

***significant at the 0.001 level (2-tailed).

**Fig. 2:**
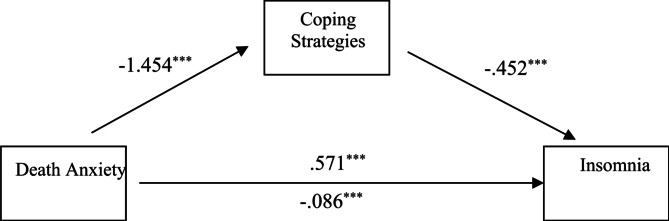
The mediation effect of coping strategies between death anxiety and Insomnia in male. ***significant at the 0.001 level (2-tailed).

**Fig. 3:**
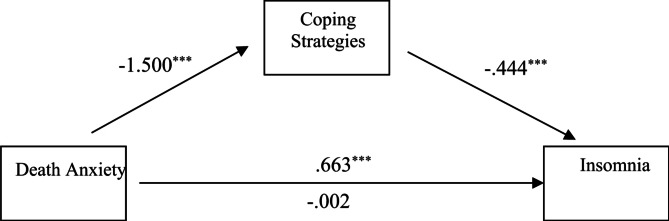
The mediation effect of coping strategies between death anxiety and Insomnia in female. ***significant at the 0.001 level (2-tailed).

Table 3; Figs. 2 and 3, show the results of regression analysis with coping strategies as the mediator variable and Insomnia as an outcome variable, with the aim of investigating the influence of coping strategies in the relationship between death anxiety (as independent variable) with Insomnia (dependent variable) between male and female respondents.

From the results, we see that for male respondents’ death anxiety was significantly and negatively associated with coping strategies (β = -1.1454, from path *a*). Similarly, coping strategies were significantly and negatively associated with Insomnia (β = -0.452, from path b). As for the direct effect of death, anxiety was significantly and positively associated with Insomnia (β = 0.571, from path *c*). When coping strategies were involved in the model as a mediator, the indirect pathway between death anxiety and Insomnia became statistically insignificant (β = -0.086, from path *c’*) which showed that coping strategies fully mediate the relationship between death anxiety and Insomnia in male respondents.

The regression analysis demonstrates that coping strategies fully mediate the relationship between death anxiety and Insomnia for both male and female respondents. This suggests that the use of coping strategies can effectively diminish the direct impact of death anxiety on insomnia, highlighting their pivotal role in managing psychological distress linked to death anxiety. Findings showed that coping strategies fully mediate the relationship between death anxiety and Insomnia in male participants. Findings also follow our results that in the clinical population psychological distress and sleep disturbances identified with direct positive relationship effect, furthermore coping strategies exhibit mediating effect^26^. Another study was conducted to assess the mediating role of coping skills in life and the workplace to reduce stress and anxiety in patients as well as play an important role in improving sleep quality^27,28^.

For female respondents’ death anxiety was significantly and negatively associated with coping strategies (β=-1.500, from path *a*). Similarly, coping strategies were significantly and negatively associated with Insomnia (β=-0.444, from path b). As for the direct effect of death, anxiety was significantly and positively associated with Insomnia (β = 0.663, from path *c*). When coping strategies were involved in the model as a mediator, the indirect pathway between death anxiety and Insomnia became statistically insignificant (β=-0.002, from path *c’*) which showed that coping strategies fully mediate the relationship between death anxiety and Insomnia in female respondents.

To evaluate the effect size of the mediating pathway, we calculate the proportion of the total effect of death anxiety on Insomnia by coping strategies using the formula *(a × b)*. For male respondents, the proportion of coping strategies mediation was 65.7% and for female respondents, the proportion of coping strategies mediation was 66.6%. this shows that for male respondents coping strategies play a higher level of mediation than that of female respondents.

The full mediation highlights that effective coping strategies can completely buffer the direct impact of death anxiety on insomnia. A study revealed the findings in the same way gender perspective is considered important and findings suggested that females tend to have more risk of developing Insomnia due to death anxiety and emotion-focused coping styles play a crucial role in managing the symptoms^29^.

**Table 4:** The comparison of death anxiety, insomnia, and coping strategies between male and female respondents.

Variables	M ± SD		t	*p*	Cohen’s d	95% CI	
	Male (*n* = 142)	Female (*n* = 122)				LL	UL
Death anxiety	14.84 ± 3.14	15.76 ± 3.21	-2.321	0.021	0.29	-1.68	-0.14
Insomnia	11.61 ± 3.04	12.66 ± 3.05	-2.797	0.006	0.34	-1.79	-0.31
Coping strategies	71.23 ± 5.90	69.20 ± 5.57	2.868	0.004	0.35	0.64	3.43

Table 4, represents results for the comparison of death anxiety, insomnia, and coping strategies between male and female respondents. From the results, we conclude that the levels of death anxiety and Insomnia are significantly higher in female respondents than in male patients with p-values, of 0.021 and 0.006 respectively while the level of coping strategies is higher in male respondents than that of female respondents with p-value 0.004.

A Study^30^ also showed similar results that a higher risk for Insomnia and death anxiety was reported in females 1.6 times as compared to males. Another finding suggested that both genders showed a high risk of Insomnia due to death anxiety both male and female, while a multivariate approach showed a high risk of Insomnia in females^29^. In a similar vein gender differences in coping styles have mixed findings. Different studies showed a greater amount of coping mechanisms used by males to regulate their emotions whereas females tend to use maladaptive coping^31–34^.

**Table 5:** The comparison of death anxiety, insomnia, and coping strategies between respondents belonging to urban and rural areas.

Variables	M ± SD		t	*p*	Cohen’s d	95% CI	
	Urban (*n* = 170)	Rural (*n* = 94)				LL	LL
Death anxiety	14.89 ± 2.79	15.91 ± 3.74	-2.511	0.013	0.31	-1.82	− 0.22
Insomnia	11.94 ± 2.93	12.39 ± 3.34	-1.157	0.248	0.14	-1.24	0.32
Coping strategies	71.09 ± 5.34	68.85 ± 6.41	3.032	0.003	0.38	0.78	3.69

Table 5, represents a comparison of death anxiety, Insomnia, and coping strategies between respondents belonging to urban and rural areas. From the results, we conclude that the level of death anxiety is higher in respondents belonging to rural areas than that of urban areas with a p-value of 0.013 while the level of coping strategies is higher in respondents belonging to urban areas than that of rural areas with a p-value of 0.003. Furthermore, no significant differences were found in the level of Insomnia between respondents belonging to urban and rural areas with a p-value of 0.248.

Previous studies with similar findings revealed that patients from urban and rural areas tend to show variations in death anxiety feelings as individuals from urban areas experienced more death anxiety as compared to rural areas^35^. Research showed similar findings and reported Insomnia and sleep-related issues were more prevalent in urban residents than in rural when treated at medical outpatients’ clinics and explored that people in rural areas use more coping strategies as compared to urban^36^.

**Table 6:** The comparison of death anxiety, insomnia, and coping strategies between respondents among different age groups.

Variable	M ± SD				F	*P*
	< 25 years (*n* = 86)	25–35 (*n* = 88)	35–45 (*n* = 60)	> 45 (*n* = 30)		
Death anxiety	16.151 ± 3.28	15.421 ± 3.70	14.267 ± 2.26	15.258 ± 1.97	5.620	< 0.001
Insomnia	12.965 ± 3.24	11.727 ± 3.73	11.683 ± 1.99	11.533 ± 1.57	3.483	< 0.016
Coping strategies	68.442 ± 6.00	70.818 ± 6.99	71.433 ± 3.81	71.767 ± 3.24	4.726	< 0.003

**Table 7:** Post-hoc (LSD) analysis for death anxiety, insomnia, and coping strategies between respondents among different age groups.

Variable	< 25 vs. 25–35	< 25 vs. 35–45	< 25 vs. > 45	25–35 vs. 35–45	25–35 vs. > 45	35–45 vs. > 45	95% CI	
							L.L	L.L
Death Anxiety	0.123	< 0.000	< 0.003	< 0.028	0.065	0.924	14.870	15.645
Insomnia	< 0.008	< 0.013	< 0.027	0.931	0.763	0.826	11.725	12.472
Coping Strategies	< 0.006	< 0.002	< 0.006	0.520	0.433	0.794	69.585	70.998

Tables 6 and 7, presents a comparison of death anxiety, Insomnia, and coping strategies among different age groups. From the results, we conclude that the levels of death anxiety, Insomnia, and coping strategies are significantly different among different age groups of the respondents with p-values of 0.001, 0.016, and 0.003 respectively. Furthermore, maximum differences were found between age group “up to 25 years” and “35–45 years” for death anxiety with a p-value of 0.000, age group “up to 25 years” and “25–35 years” for Insomnia with a p-value 0.008 and age group “up to 25 years” and “35–45 years” for coping strategies with p-value 0.002.

Previous studies support the findings that death anxiety is usually reported high in young patients and it reduced with age as in the elderly. In contrast, Insomnia increases with age in most of the patients^37^. Another study explored that older adults are less likely to use coping mechanisms than younger adults^38^. Similar findings showed] that death-related anxiety peaked in both genders during their adulthood and significantly declined thereafter^39^. Thus, the use of coping strategies are important protective factor for the management of death anxiety and Insomnia in patients.

## Conclusion

In the present study, we examined death anxiety and Insomnia in the clinical population and the mediating effect of using coping strategies from a gender perspective. In conclusion, the present study provides evidence in the light of literature as well as statistical findings about the significant positive relationship between death anxiety and Insomnia, and both variables showed a negative relationship with coping strategies. The findings reveal significant gender-based differences in death anxiety, insomnia, and coping strategies. Female respondents report higher levels of death anxiety and Insomnia compared to male respondents. Females also tend to use more emotion-focused coping strategies, whereas males favor problem-focused coping. Furthermore, coping strategies are also concluded to mediate the role of death anxiety in Insomnia. Understanding the factors that mediate the relationship between death anxiety and Insomnia is crucial for developing effective psychological interventions.

Research regarding this is somehow limited as this concept is in the phase. Sample taken to determine the role of death anxiety in Insomnia was taken at a small scale i.e. from hospitals in one city only. The present study checks the role of death anxiety in a limited number of diseases. The influence of demographic variables on the severity of psychiatric disorders is not examined in this study. The study may rely on self-report questionnaires or scales to assess death anxiety, coping strategies, and Insomnia, which can be subject to response biases or influenced by participants’ subjective interpretations. Without a control group of individuals without non-communicable diseases, it may be difficult to attribute the observed relationships specifically to the presence of these diseases.

The role of death anxiety has been examined very rarely till now. It is suggested to be analyzed with more mediating and moderating variables. Constructs, one as death anxiety are suggested to be studied on other psychiatric and mental disorders. Investigate other potential moderating or mediating factors, such as social support, disease severity, or psychological well-being that may influence the relationships between death anxiety, coping strategies, and Insomnia in this population. Incorporate qualitative methods, such as interviews or focus groups, to gain a deeper understanding of the lived experiences, perceptions, and coping mechanisms of patients with non-communicable diseases, and how these may differ by gender.

## Data Availability

The data supporting this study’s findings are available from the corresponding author, (syg@sdu.edu.cn) upon reasonable request.
